# Application of MALDI-TOF MS Profiling Coupled With Functionalized Magnetic Enrichment for Rapid Identification of Pathogens in a Patient With Open Fracture

**DOI:** 10.3389/fchem.2021.672744

**Published:** 2021-04-30

**Authors:** Jichong Ying, Wenjing Gao, Dichao Huang, Chuanfan Ding, Ling Ling, Tao Pan, Shaoning Yu

**Affiliations:** ^1^ Ningbo No. 6 Hospital, Ningbo, China; ^2^ Key Laboratory of Advanced Mass Spectrometry and Molecular Analysis of Zhejiang Province, Institute of Mass Spectrometry, School of Material Science and Chemical Engineering, Ningbo University, Ningbo, China; ^3^ Department of Breast Surgery and Oncology, The Second Affiliated Hospital, Zhejiang University School of Medicine, Hangzhou, China

**Keywords:** MALDI-TOF MS, orthopedic trauma infection, magnetic enrichment, pathogen identification, *Vibrio alginolyticus*

## Abstract

Posttraumatic infections can occur in orthopedic trauma patients, especially in open fractures. Rapid and accurate identification of pathogens in orthopedic trauma is important for clinical diagnosis and antimicrobial treatment. Matrix-assisted laser desorption/ionization time-of-flight mass spectrometry (MALDI-TOF MS) has been successfully used for first-line identification of pathogens grown on culture plates. However, for direct analysis of liquid clinical specimens, pre-purification of the sample is necessary. Herein, we investigated the feasibility of coupling Fc-MBL@Fe_3_O_4_ enrichment with MALDI-TOF MS profiling in the identification of pathogens in liquid-cultured samples. This method is successfully used for the identification of pathogens in a patient with an open-leg fracture obtained at sea. Pathogens were enriched by Fc-MBL@Fe_3_O_4_ from briefly pre-cultured liquid media and identified by MALDI-TOF MS. We identified an opportunistic pathogen, *Vibrio alginolyticus*, which is uncommon in clinical orthopedic trauma infection but exists widely in the sea. Therefore, combining Fc-MBL@Fe_3_O_4_ enrichment and MALDI-TOF MS profiling has great potential for direct identification of microbes in clinical samples.

## Introduction

Orthopedic traumas are complex and diverse. Serious wounds are always difficult to debride; especially, when complicated fractures are combined, the surgical treatment is often required (Morgenstern et al., 2018; Tuon et al., 2019). Posttraumatic infections can occur in orthopedic trauma patients when it is not treated in a timely manner or after surgical treatment (Arnold et al., 2013; Yun et al., 2016; Backes et al., 2018). Rapid identification of pathogenic bacteria in orthopedic trauma, especially open fractures, guides the clinical diagnosis and antimicrobial treatment (Yun et al., 2016; Patrulea et al., 2020). Thus, superficial wound swabs or deep fluid samples are always sent to the clinical microbiology lab for bacterial identification.

The current standard of bacterial identification in hospitals continues to rely on culture-based biochemical testing, which may take days to complete (Varadi et al., 2017; Backes et al., 2018). Moreover, culture-based methods suffer increasing skepticism about their sensitivity and accuracy (Rhoads et al., 2012; Firoozabadi et al., 2015). Molecular methods that rely on the analysis of genomic markers, such as ribosomal RNA sequencing, have better sensitivity and accuracy for pathogen identification (Rhoads et al., 2012). However, molecular diagnostic methods have high cost and require sophisticated expertise (Woo et al., 2008; Segawa et al., 2014). In recent years, matrix-assisted laser desorption/ionization time-of-flight mass spectrometry (MALDI-TOF MS) has been introduced into clinical microbiology laboratories. Due to the specificity, speed of analysis, and low cost of consumables, MALDI-TOF MS has been widely acclaimed for clinical bacterial identification (Welker et al., 2019; Feng et al., 2020; Nomura et al., 2020; Papagiannopoulou et al., 2020). MALDI-TOF MS identification is mainly based on the MS fingerprint pattern of bacterial ribosomal proteins in the *m/z* range of 2–20 kDa, which can be compared to the MS database to identify the bacterial genus and species (Singhal et al., 2015). MALDI-TOF MS databases with more than 4000 strains have been set up and are widely used in the identification of clinical bacteria, fungi, mycobacteria, and *Nocardia* (Feng et al., 2020). Nevertheless, the single colonies are always needed for this method. Clinical samples or liquid cultured samples are inoculated on solid culture plate to grow single colonies for identification by MALDI-TOF MS. For direct analysis of bacteria in liquid clinical samples, the pre-purification steps are mandatory. Since approximately 10^5^ CFU of bacteria are needed for successful identification, direct analysis of bacteria in clinical samples seems only possible for urine samples (Zboromyrska et al., 2016). Utilizing of liquid cultures has increased the sensitivity and decreased the turn-around time of bacterial culture, especially for samples with low bacterial loads. Hence, development of pre-purification methods for liquid-cultured bacterial sample is sorely needed.

Functionalized magnetic nanoparticles (MNPs) have been developed to capture bacteria and simplify the purification processes due to its large ratio of surface area to volume, ease of operation, and good biocompatibility (Liébana et al., 2009; Cheng et al., 2016; Zhu et al., 2016; Yi et al., 2018; Nemr et al., 2019). Specific aptamers and antibodies were conjugated with MNPs to enrich certain bacteria or part of microbes from suspension (Liébana et al., 2009; Cheng et al., 2016; Zhu et al., 2016; Yi et al., 2018; Nemr et al., 2019). Mannose-binding lectin (MBL) is an important opsonin component of the lectin pathway associated with innate immunity, which can recognize and bind carbohydrates on the surfaces of different bacteria, fungi, and viruses in a calcium-dependent manner (Takahashi and Ezekowitz, 2005). Ingber et al. genetically engineered a new version of MBL by fusing the carbohydrate recognition region of MBL to the flexible neck of the Fc portion of IgG1, which conjugated with MNPs and was used to cleanse septic blood or enrich bacteria in clinical samples (Kang et al., 2014; Bicart-See et al., 2016). These studies indicated that Fc-MBL has binding capability for a wide range of microbes, which prompted us to utilize Fc-MBL@Fe_3_O_4_ to enrich bacteria and combine with the MALDI-TOF MS identification.

**SCHEME 1:**
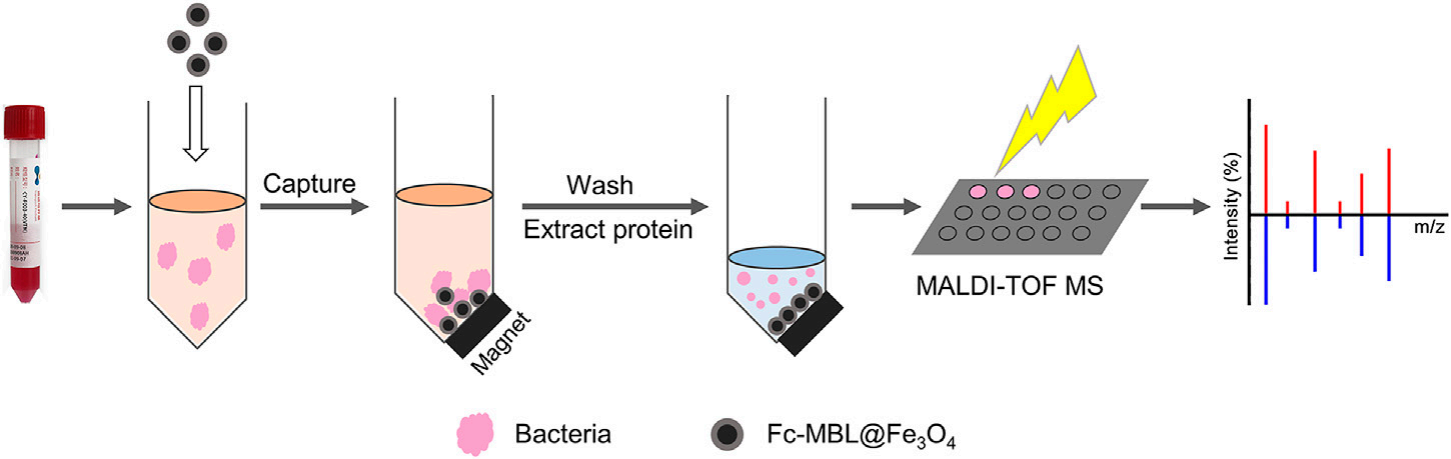
Workflow for the identification of pathogens from the clinical liquid sample.

Herein, we investigated the feasibility of the MALDI-TOF MS profiling coupled with Fc-MBL@Fe_3_O_4_ enrichment for identification of bacteria in liquid culture media (Scheme 1). After the verification, the application of this method in real case was explored. A patient obtained an open fracture at sea and did not acquire treatment for more than 20 h due to diagnostic limitations. Considering that the patient was injured at sea, uncommon species of bacteria may have adhered to the wound; we attempt to use Fc-MBL@Fe_3_O_4_ enrichment coupled with MALDI-TOF MS identification. A wound swab and fluid were short-term cultured on liquid media and analyzed by the proposed method, wherein *Vibrio alginolyticus* was identified. These results show that these pathogen identification procedures are more rapid than traditional methods and beneficial for clinical treatment of patients.

## Material and Methods

### Patient

A 56-year-old male patient was crushed while working on the boats, and his right calf was stressed, accompanied by skin lacerations and bleeding. He had an open wound approximately 6 × 15 cm in the lower part of the right calf, and the fractured end was exposed. Because the patient was unable to acquire clinical treatment at sea, the wound was simply bandaged to stop the bleeding. After more than 20 h, the patient was sent to the hospital and diagnosed with open comminuted fracture of the lower right tibia and fibula. This study was approved by the Ethics Committee of Ningbo No. 6 Hospital.

### Chemicals and Instruments

*α*-Cyano-4-hydroxycinnamic acid (CHCA), formic acid, and acetonitrile (ACN) were purchased from Merck (Darmstadt, Germany). Trifluoroacetic acid (TFA) was obtained from Shanghai Macklin Biochemical Technology Co., Ltd. (Shanghai, China). Tryptone soybean agar (TSA) and Luria–Bertani (LB) broth were purchased from Beijing Land Bridge Technology Co., Ltd (Beijing, China). The functionalized MNPs (Fc-MBL@Fe_3_O_4_) were synthesized as in our previous study (Sun et al., 2021). The detailed synthesis procedures and characterization results are presented in the Supporting Information.

The mass spectra for the verification of method were obtained by Autoflex max TOF/TOF mass spectrometer (Bruker Daltonics, Germany) with a pulsed Nd:YAG laser (355 nm) in a linear positive mode. The acceleration voltage was set at 20 kV, each spectrum was acquired by 1000 laser shots, and the laser intensity was regulated to ensure a good signal-to-noise ratio. MALDI-TOF MS identification was carried out using an M-Discover 100 mass spectrometer (Zhuhai Meihua Medical Technology Co., Ltd. China) in a linear positive mode with the *m/z* range 2–20 KDa. The mass spectra were calibrated by *Escherichia coli* (ATCC 8739) according to the manufacturer’s instructions. MicroCtrl 1.0 software was used to acquire the spectra and for real-time interpretation and identification; the raw spectra were processed by smoothing, baseline removal, and peak-picking, and the processed spectra were compared with the reference spectra in the database by using the pattern-matching algorithm of the software. The scores that ranged from 0.00 to 3.00 were calculated based on the correlation between the two spectra. According to the manufacturer, a score >2.0 is considered reliable in the species level, a score 1.7–2.0 indicates identification in the genus level, and a score <1.7 indicates unreliable result. Transmission electron microscopy (TEM) images were obtained by microscope JEOL JEM 2100 (Japan) at 200 kV.

### Sample Preparation and MNP Enrichment

For verification of the method, the standard bacteria strains (*S. aureus*, ATCC 25923; *K. pneumoniae*, CICC 21519) were inoculated on the TSA solid plate, and the single colonies were adjusted to certain concentrations by measuring the absorbance at 600 nm using UV–vis absorption spectroscopy. For the enrichment efficiency of Fc-MBL@Fe_3_O_4_, 1000 or 100 CFUs of *S. aureus* and 10 μl of Fc-MBL@Fe_3_O_4_ solution (10 mg/ml) were added in 30 μl Tris-HCl buffer (0.1 mM, pH 7.4) containing 10 mM CaCl_2_ and 0.05% Tween-20 and incubated at 37°C for 15 min with shaking. After magnetic enrichment, the pellets were washed twice with 30 μl of water. The supernatant was collected and cultured on the TSA solid media for 13 h, together with the original solution and pellets. For the *S. aureus* assay, different amounts (10^8^, 10^7^, 10^6^, and 10^5^ CFU) of bacteria in 1 ml of buffer solution were enriched by the similar method, wherein the pellets were washed with 100 μl of water. The *S. aureus* and *K. pneumoniae* in LB liquid media at the concentration of 10^8^ CFU/ml were carried out as follows; 300 μl of buffer solution and 10 μl of Fc-MBL@Fe_3_O_4_ solution were added subsequently in 100 μl of bacteria suspension, and the mixture was incubated at 37°C for 15 min with shaking. The pellets were washed twice with 100 μl of water. The bacterial proteins were extracted from the Fc-MBL@Fe_3_O_4_ by use of 5 μl of 70% formic acid and 5 μl of ACN, and the extracted solution was subjected to MALDI-TOF MS analysis.

For the real case, a wound swab and 1 ml of trauma fluid from the patient were collected in sterile containers and subjected to magnetic enrichment and MALDI-TOF MS profiling. The wound swab and fluid were both subcultured in 5 ml of liquid LB medium at 37°C for 8 h. For enrichment, 1 ml of buffer solution was added to 300 μl of subcultured liquid, and then, 20 μl of Fc-MBL@Fe_3_O_4_ solution was added. The obtained solution was incubated at 37°C for 15 min with shaking. After magnetic enrichment, the pellets were washed twice with 100 μl of water. The bacterial proteins were extracted as above description. For the verification, the subcultured liquid samples were grown on the TSA solid medium for 24 h, and the bacterial colonies were identified by MALDI-TOF MS.

### MALDI-TOF MS Analysis

The bacterial protein solution (1 μl) extracted from MNPs was deposited on a MALDI target plate. Then, CHCA matrix (10 mg/ml in ACN/H_2_O (v/v = 1/1) containing 2% TFA) was deposited on the sample spot after drying. For analysis of bacteria grown on the solid TSA medium, the standard extraction method was used (Matsuda et al., 2012). Briefly, a 1-μl loop of bacteria was suspended in 300 μl H_2_O, and 900 μl ethanol was added. After vigorous vortex, the mixture was centrifuged at 12,000 rpm for 5 min. The supernatant was discarded, and 30 μl of 70% formic acid was added to the pellets and thoroughly mixed. Next, 30 μl of ACN was added, and the resulting solution was centrifuged again at 12,000 rpm for 5 min. One microliter of supernatant was deposited on the MALDI target plate and dried at room temperature. Finally, 1 μl of CHCA matrix was placed on the sample spots and left to dry.

### Clinical Routine Pathogen Identification

The clinical samples were inoculated on the Columbia blood agar, Sabourand’s fungus agar, and anaerobic medium plate in a sterile manner and incubated in 35 ± 2°C for 24–48 h in clinical microbiology laboratory. The bacteria grown on the solid media were subjected to Gram staining, and the isolates were identified using a VITEK2-Compact automatic bacterial identification instrument (Biomérieux) in accordance with the manufacturer’s instructions.

## Results and Discussion

### Feasibility of the Proposed Method

**FIGURE 1:**
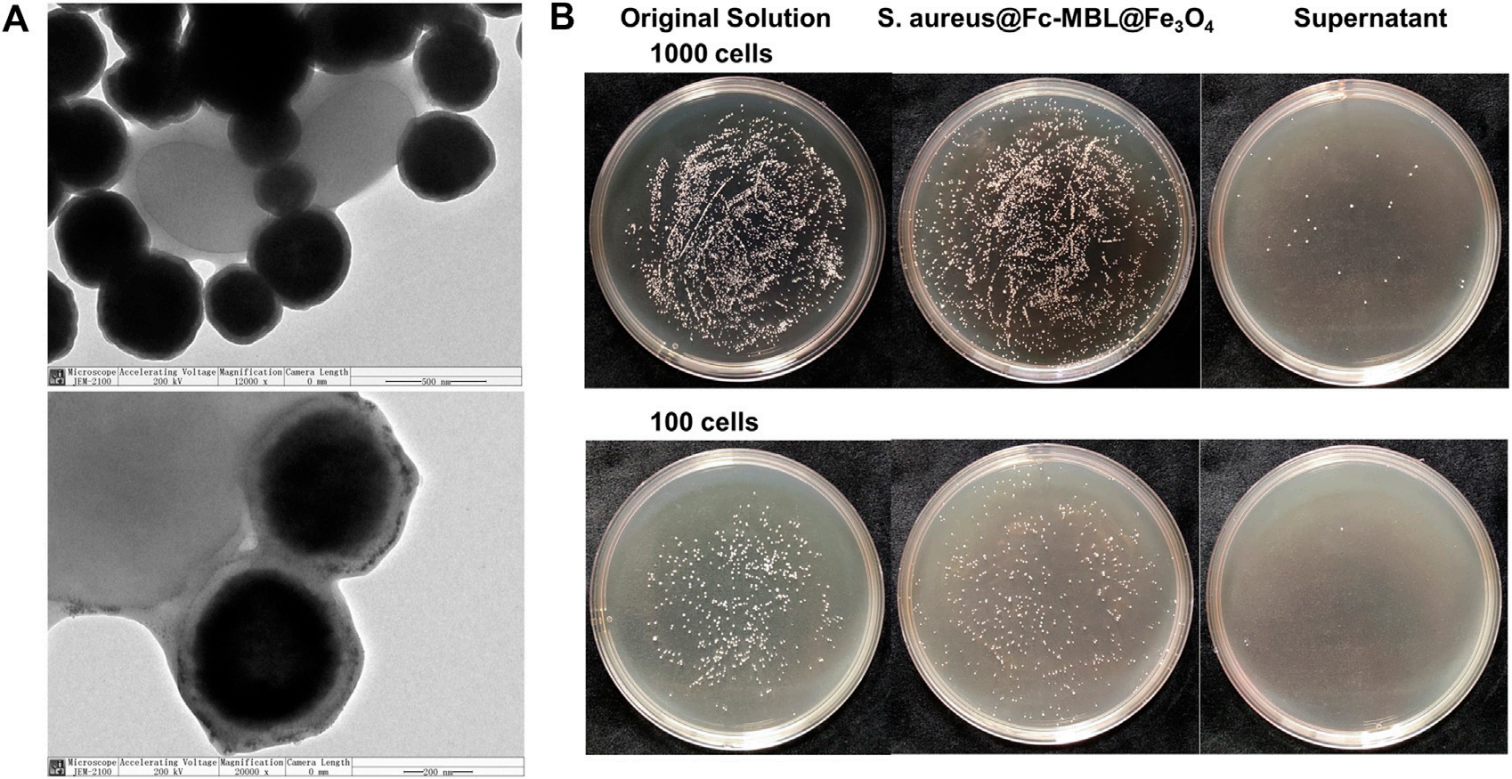
**(A)** TEM images of *S. aureus* conjugated with Fc-MBL@Fe_3_O_4_. **(B)** Photographs of cultured plate of original solution, enriched pellets, and supernatant.

**FIGURE 2:**
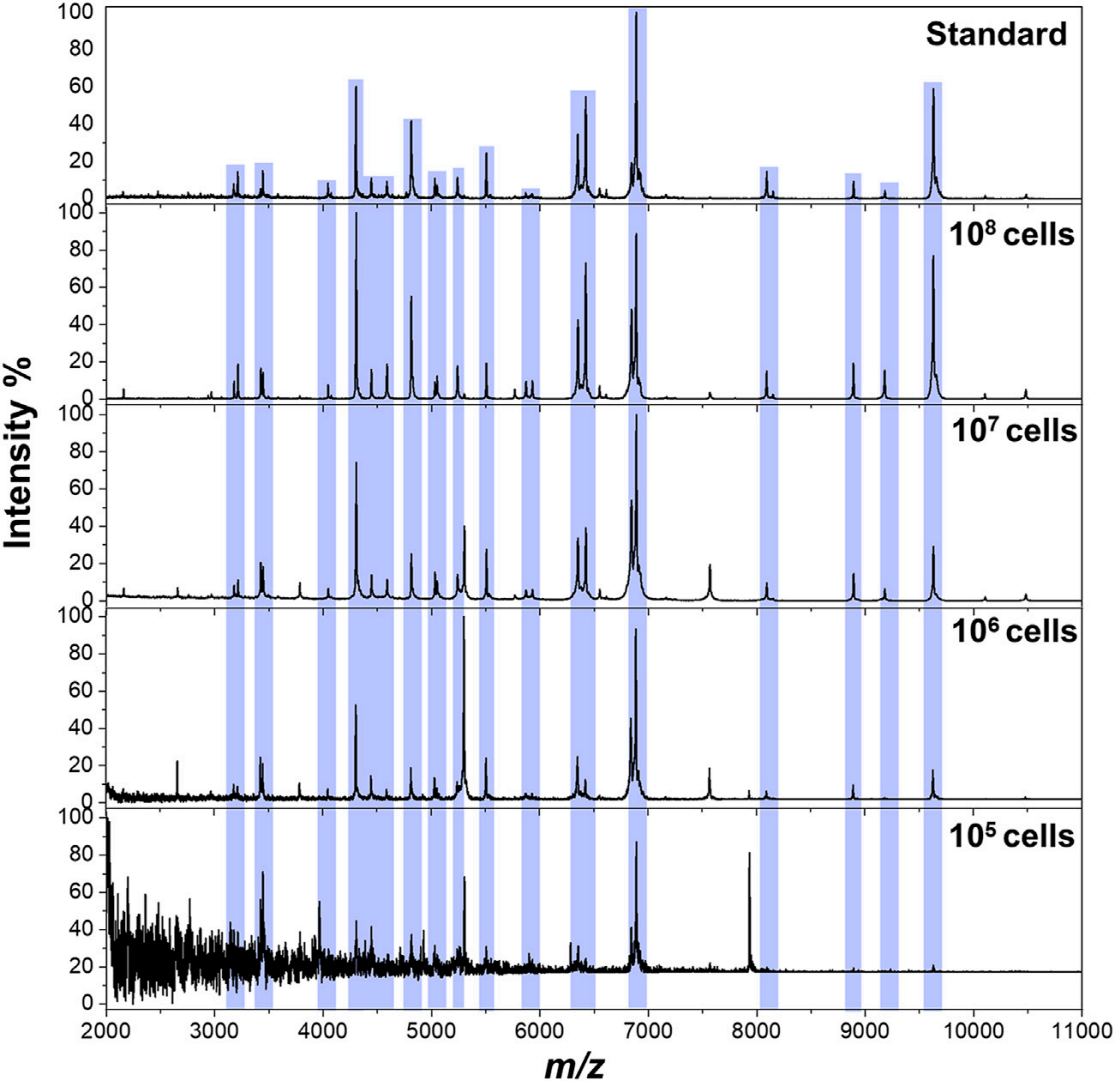
MALDI-TOF MS spectra of *S. aureus* obtained from pure solution **(top)** and enriched by Fc-MBL@Fe_3_O_4_ from 1 ml buffer solution at different amounts.

The workflow of the proposed method was illustrated in Scheme 1. The pathogens in liquid clinical samples or short-term–cultured liquid samples were captured by Fc-MBL@Fe_3_O_4_; the enriched bacteria were extracted by formic acid from Fc-MBL@Fe_3_O_4_ and subjected to MALDI-TOF MS identification. Before application in real case, we investigated the feasibility of the method. A common pathogen in orthopedic infection, *Staphylococcus aureus* (*S. aureus*), was selected as model to verify the method. The synthesis and characterization of Fc-MBL@Fe_3_O_4_ were presented in supporting information. The TEM images of Fc-MBL@Fe_3_O_4_ binding to *S. aureus* are shown in Figure 1A, which indicated the binding capability of Fc-MBL@Fe_3_O_4_ to *S. aureus*. Next, the capture efficiency of Fc-MBL@Fe_3_O_4_ was evaluated by the plate-counting method. Briefly, the different amounts of *S. aureus* suspension were enriched by Fc-MBL@Fe_3_O_4_; the bacteria in original solution, captured by Fc-MBL@Fe_3_O_4_, and the supernatant were inoculated on the TSA plate in parallel, and the results are shown in Figure 1B. It can be seen that bacteria were almost captured completely by Fc-MBL@Fe_3_O_4_. Then, the different amounts (10^8^/10^7^/10^6^/10^5^ CFU) of *S. aureus* in 1 ml buffer solution were enriched by Fc-MBL@Fe_3_O_4_ and identified by MALDI-TOF MS. As shown in Figure 2, the spectra of ≥10^6^ CFU of bacteria in buffer enriched by Fc-MBL@Fe_3_O_4_ show similar profiles with the standard spectrum of pure bacteria. It is should be noted that approximately ∼10^5^ CFU are needed on the target plate for successful bacteria identification with MALDI-TOF MS (Segawa et al., 2014), which means the concentration of 10^8^ CFU/ml is needed, wherein 1 μl of bacteria suspension is deposited on the target plate. In this method, the enriched pellets were eluted by 10 μl solution (5 μl 70% formic acid +5 μl ACN), and the 1 μl of solution is deposited on the target plate. Thus, the results are rational that 10^6^ CFU of bacteria in 1 ml buffer solution could be detected successfully by the proposed method.

**FIGURE 3:**
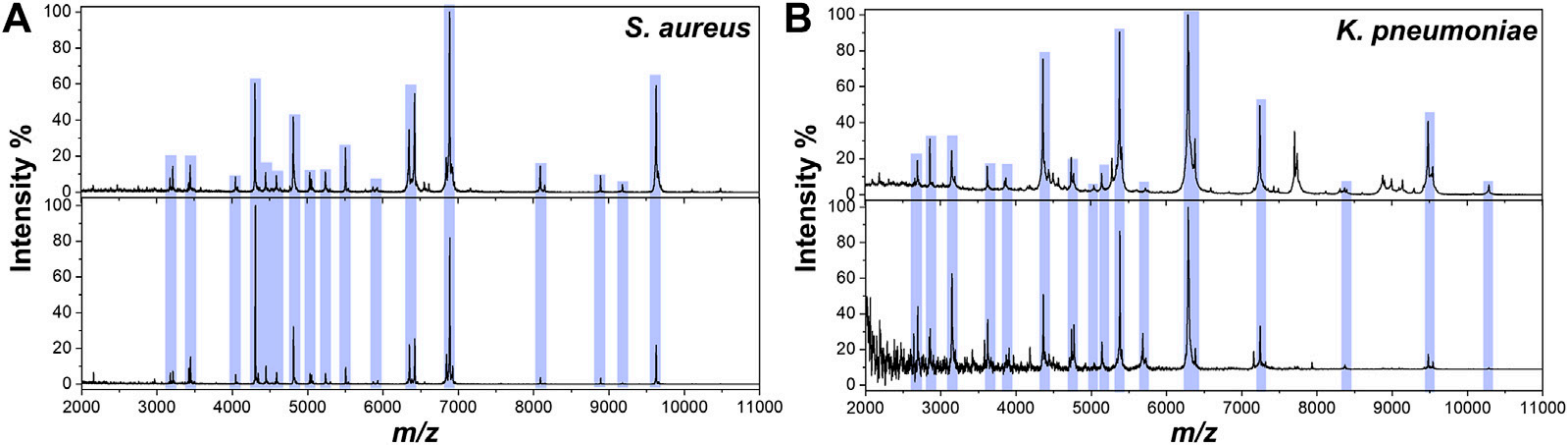
MALDI-TOF MS spectra of **(A)** *S. aureus* and **(B)** *K. pneumoniae* obtained from pure solution **(top)** and enriched by Fc-MBL@Fe_3_O_4_ from LB liquid media **(bottom)**.

Furthermore, *S. aureus* and another bacteria *Klebsiella pneumoniae* (*K. pneumoniae*) were spiked in LB liquid media at 10^8^ CFU/ml (the concentration of clinical positive cultures is ∼10^8^ CFU/ml). The 100 μl of liquid bacterial cultures were enriched by Fc-MBL@Fe_3_O_4_ and analyzed by MALDI-TOF MS. The pure bacteria were tested by MALDI-TOF MS, and the obtained spectra were compared with those captured by Fc-MBL@Fe_3_O_4_ from LB media. As shown in Figure 3, the spectra of captured bacteria (*S. aureus* and *K. pneumoniae*) from LB media show similar profiles to pure bacteria. For evaluation of reproducibility, the sample of *S. aureus* in LB broth was carried out in triplicate, and each sample was deposited at three spots; the obtained spectra all show matched profiles (Supplementary Figure S5). These results indicated the feasibility of the proposed method for rapid identification of bacteria in clinical bacterial liquid cultures.

### Identification of Bacteria in a Patient With Open Fracture

**FIGURE 4:**
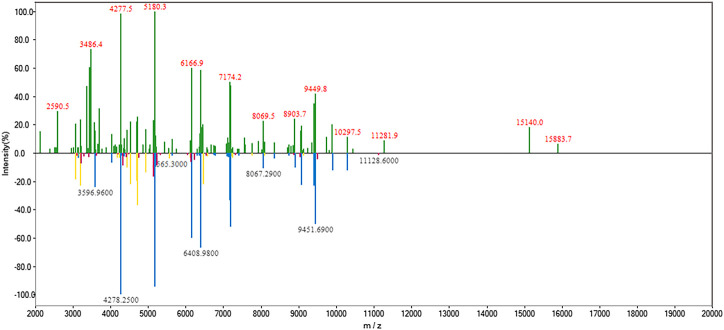
MALDI-TOF MS spectrum of bacteria enriched by Fc-MBL@Fe_3_O_4_ **(top)** and matched spectrum of *V. alginolyticus* in the database **(bottom)**. In the lower spectrum, the blue peaks represent matched peaks with high similarity, red peaks represent low similarity, and yellow peaks represent intermediate similarity.

Rapid and accurate identification of progressive bacteria colonizing orthopedic trauma, especially open fracture, is important for clinical diagnosis and treatment to avoid posttraumatic or postoperative infections. In the case presented here, the patient’s leg suffered an open fracture on the coastal sea of China. Fc-MBL@Fe_3_O_4_ possesses a universal enrichment capacity for different pathogens, and MALDI-TOF MS can identify a wide range of pathogens, including bacteria and fungi, with high accuracy at both the genus and species level (97–99% and 85–97%, respectively) (Patel, 2013). Considering the special of this case, we attempt to use MALDI-TOF MS in coupling with Fc-MBL@Fe_3_O_4_ enrichment for the identification of probable pathogens in this case. For analysis of clinical bacterial samples, a short-term subculture step is always needed since the amount of ∼10^5^ CFU is requisite for MALDI-TOF MS identification. Herein, the wound swab and fluid were short-term cultured in liquid LB media, and the liquid samples were analyzed by the proposed method. As a control, a sterile swab was cultured in liquid LB media in parallel. Four sample spots were tested in parallel, and the representative mass spectrum is shown in Figure 4. The mass spectra of the bacteria in the wound swab matched *Vibrio alginolyticus* (*V. alginolyticus*), a common pathogenic bacteria of numerous aquatic animals that is widely present in the sea, with an average score of 2.15 (Table 1). The fluid sample was also identified as *V. alginolyticus*, with an average score of 2.31 (Table 1). Thus, these scores indicated that the identification results were reliable at the species level. The LB media of the control group keep the clear liquid phase, and the identification results by the proposed method were unreliable (score <1.7), indicating that the control group is negative for bacteria.

**TABLE 1:** MALDI-TOF MS identification of bacteria in a real case.

Sample	The proposed method					Solid culture-based method		
	Genus	Species	Score			Genus	Species	Score
Swab	*Vibrio*	*alginolyticus*		2.14		*Vibrio*	*alginolyticus*	2.41
Swab	*Vibrio*	*alginolyticus*		2.14		*Vibrio*	*alginolyticus*	2.39
Swab	*Vibrio*	*alginolyticus*		2.12		*Vibrio*	*alginolyticus*	2.27
Swab	*Vibrio*	*alginolyticus*		2.21		*Vibrio*	*alginolyticus*	2.35
Fluid	*Vibrio*	*alginolyticus*		2.29		*Vibrio*	*alginolyticus*	2.28
Fluid	*Vibrio*	*alginolyticus*		2.26		*Vibrio*	*alginolyticus*	2.29
Fluid	*Vibrio*	*alginolyticus*		2.33		*Vibrio*	*alginolyticus*	2.35
Fluid	*Vibrio*	*alginolyticus*		2.35		*Vibrio*	*alginolyticus*	2.30

### Verification of the Results

To verify the results, short-term–cultured liquid samples continue to grow on a solid medium, and the isolates were analyzed by MALDI-TOF MS. The results were consistent with those obtained by magnetic enrichment coupled with MALDI-TOF MS (Table 1 and Supplementary Figure S6).

Furthermore, Gram staining showed that the bacteria were Gram-negative rods (Supplementary Figure S7), and the pathogen was identified as *V. alginolyticus* by the VITEK2-Compact automatic bacterial identification system. *V. alginolyticus*, a type of Gram-negative opportunistic bacteria, infects both humans and aquatic animals (Xie et al., 2020). This infection is one of the main causes of aquaculture disease resulting in economic losses in mariculture in South China (Xie et al., 2005; Yu et al., 2018; Yu et al., 2019). Therefore, it was not surprising that *V. alginolyticus* was identified in this case. The whole process of the routine method required >24 h. The identification results from MALDI-TOF MS coupled with Fc-MBL@Fe_3_O_4_ enrichment were in agreement with the conventional method and required only ∼9 h.

## Conclusion

In conclusion, direct pathogen identification from liquid-cultured clinical samples can be achieved by MALDI-TOF MS identification and appropriate pre-purification. Fc-MBL@Fe_3_O_4_ could recognize and capture broad-spectrum microbes, and therefore, it is adaptable to combine with MALDI-TOF MS. In this study, we identified *V. alginolyticus* in a patient with an open fracture using MALDI-TOF MS profiling coupled with Fc-MBL@Fe_3_O_4_ enrichment. With extension of the MALDI microbial database, particularly for bacterial mixtures, magnetic enrichment coupled with MALDI-TOF MS has great potential for more clinical samples.

## Data Availability

The original contributions presented in the study are included in the article/Supplementary Material, further inquiries can be directed to the corresponding authors.
