# Supplementary material for: Application of MALDI-TOF MS Profiling Coupled With Functionalized Magnetic Enrichment for Rapid Identification of Pathogens in a Patient With Open Fracture
Source: Front Chem. 2021 Apr 30;9:672744. doi: 10.3389/fchem.2021.672744 (PMC8120279; doi:10.3389/fchem.2021.672744)
Supplement: Supplementary file 1 [file DataSheet1.docx]

**Supporting Information**

**Application of MALDI-TOF MS profiling coupled with functionalized magnetic enrichment for rapid identification of pathogens in a patient with open fracture**

**Jichong Ying^1†^, Wenjing Gao^2†^, Dichao Huang^1^, Chuanfan Ding^2^, Ling Ling^2*^, Tao Pan^3*^, Shaoning Yu^2*^**

^1^Ningbo No. 6 Hospital; ^2^Key Laboratory of Advanced Mass Spectrometry and Molecular Analysis of Zhejiang Province, Institute of Mass Spectrometry, School of Material Science and Chemical Engineering, Ningbo University, Ningbo, Zhejiang, 315211, China; ^3^Department of Breast Surgery and Oncology, The Second Affiliated Hospital, Zhejiang University School of Medicine, Hangzhou, China.

*** Correspondence:**

Dr. Ling Ling

E-mail: [lingling@nbu.edu.cn](mailto:lingling@nbu.edu.cn)

Dr. Shaoning Yu

E-mail: yushaoning@nbu.edu.cn

Dr. Tao Pan

E-mail: 2311318@zju.edu.cn

†These authors contributed equally to this work.

**Synthesis of Fc-MBL@Fe_3_O_4_**

**Materials:** Streptavidin-coated superparamagnetic nanobeads (300 nm) were purchased from Beaver Biosciences, Inc. (Suzhou, China). Phosphate buffered saline (PBS; 136.89 mM NaCl, 2.67 mM KCl, 8.24 mM Na_2_HPO_4_, 1.76 mM KH_2_PO_4_ in deionized water, pH 6.4) and pyridoxal-5-phosphatemonohydrate (PLP) were purchased from Sigma-Aldrich (St. Louis, MO, USA). Aminooxy biotin was purchased from Biotum, Inc. (Hayward, CA, USA). Polyethylene glycol (PEG)-biotin (1 kDa PEG) was purchased from Nanocs, Inc. (New York, USA).

**Synthesis of Fc-MBL@Fe_3_O_4_:** Fc-MBL@Fe_3_O_4_ was prepared as reported previously [1-3]. Fc-MBL was produced by combining the Fc region of IgG1 with the human mannose-binding lectin (MBL) with the carbohydrate recognition domain facing outward. Fc-MBL protein (4 mg/mL) was incubated with PLP (7.5 mM) in sodium phosphate buffer (50 mM, pH 6.4) overnight at room temperature. The PLP-treated protein was dialyzed three times against sodium phosphate (50 mM, pH 6.4), and then incubated overnight in the presence of 25 μg of aminooxy biotin per 1 mg of Fc-MBL. Biotinylated Fc-MBL was coupled to streptavidin-coated magnetic beads at a ratio of 25 μg protein per 1 mg of beads for 30 min at room temperature. The remaining unbound streptavidin was blocked with PEG-biotin (1 kDa PEG). The obtained Fc-MBL@Fe_3_O_4_ was stored at 4°C.

**Characterization of Fc-MBL@Fe_3_O_4_**


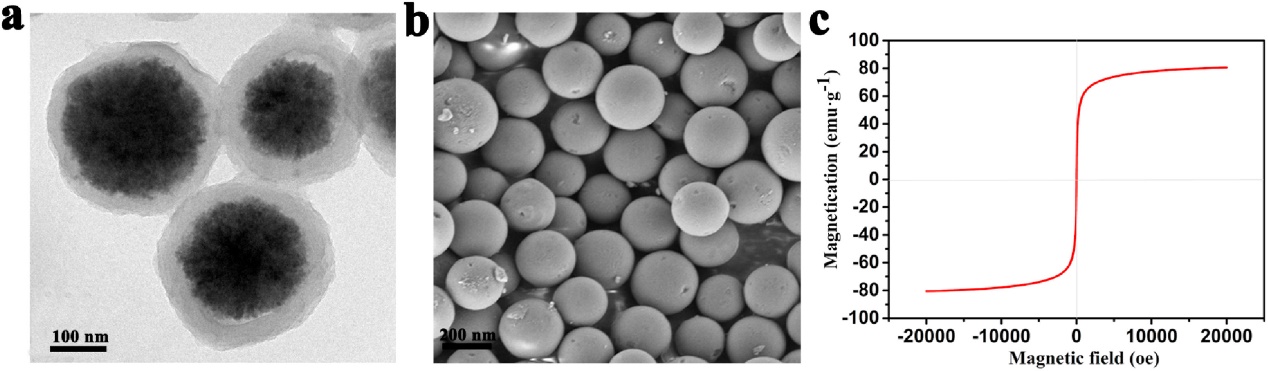


**Figure S1.** Characterization of Fc-MBL@Fe_3_O_4_ nanoparticles. (a) Transmission electron microscopy (TEM). (b) Scanning electron microscopy (SEM). (c) Magnetic hysteresis curve for the Fc-MBL@Fe_3_O_4_ nanoparticles.


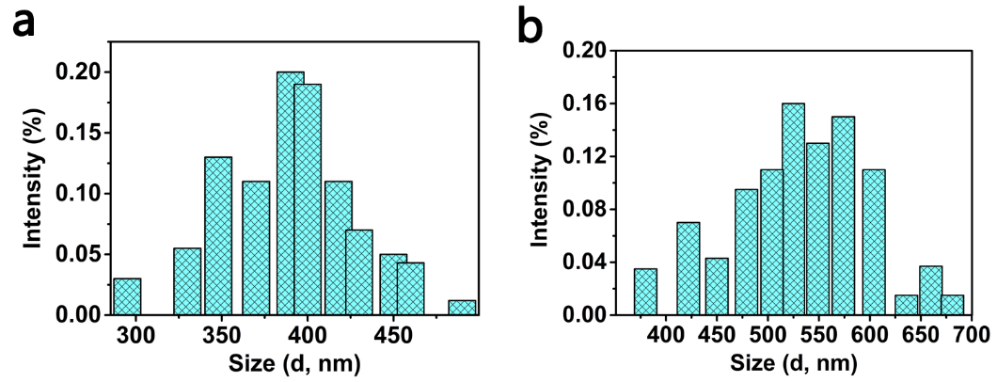


**Figure S2.** The size distribution of (a) Fe_3_O_4_ and (b) FcMBL@Fe_3_O_4_ determined by dynamic light scattering.


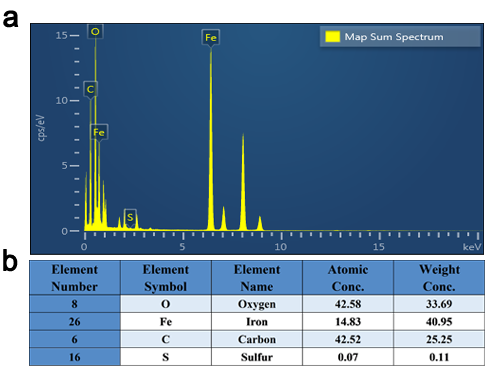


**Figure S3.** Energy dispersive X-ray (EDX) analysis of FcMBL@Fe_3_O_4_ nanoparticles.


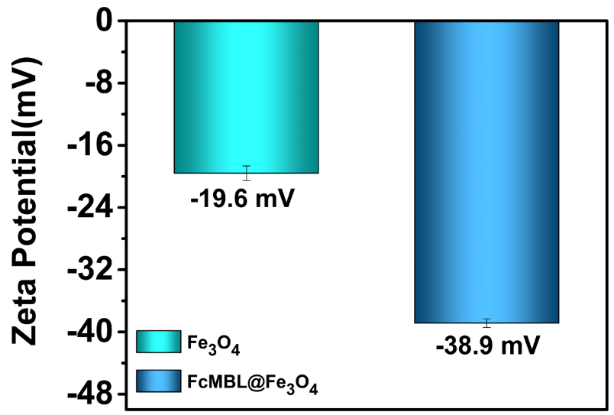


**Figure S4.** Zeta potential of Fe_3_O_4_ and Fc-MBL@Fe_3_O_4_ magnetic beads.


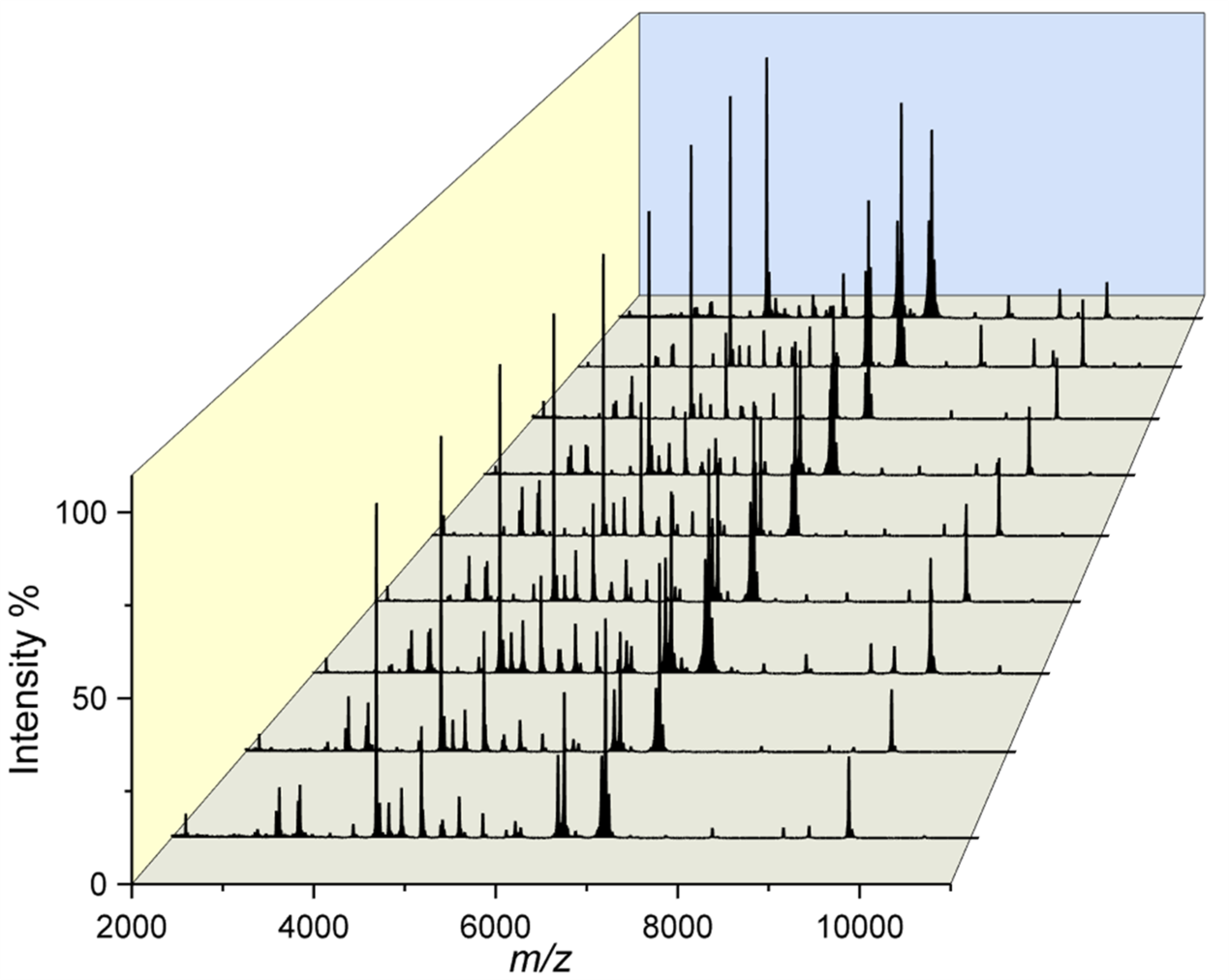


**Figure S5. MALDI-TOF MS spectra of *S. aureus* enriched from liquid LB broth by Fc-MBL@Fe_3_O_4._ (Three samples were carried out in parallel and each sample was deposited three spots.)**

**
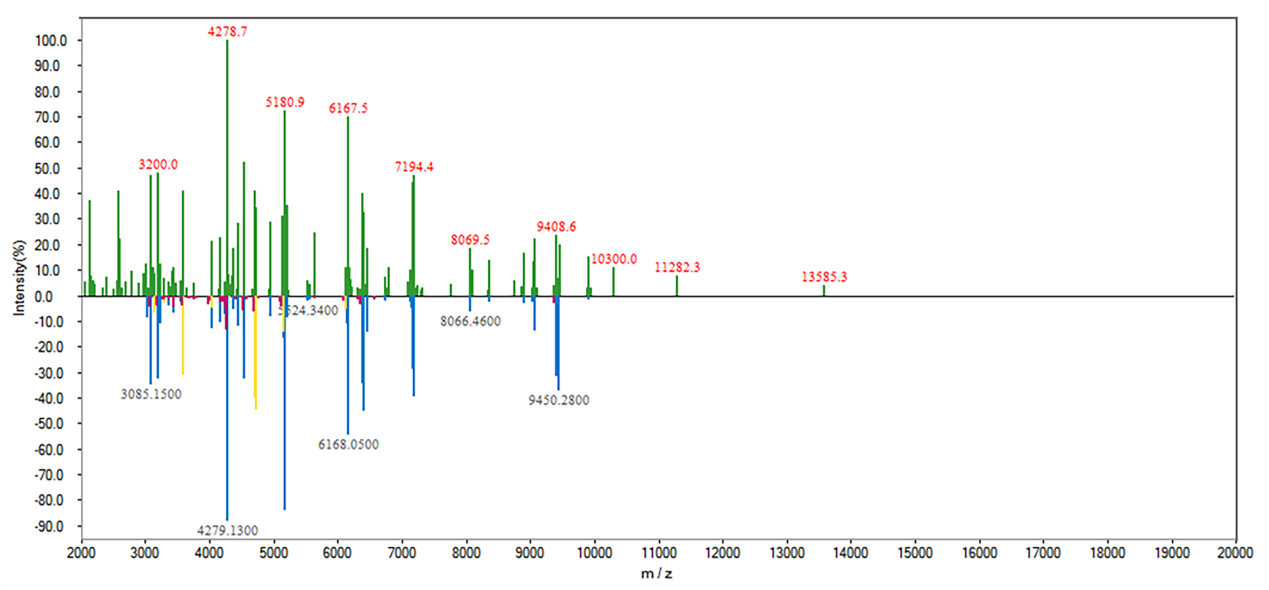
**

**Figure S6.** MALDI-TOF MS spectrum of bacteria grown on a solid plate (top) and matched spectrum of *V. alginolyticus* (bottom). In the lower spectrum, the blue peaks represent matched peaks with high similarity, red peaks represent low similarity, and yellow peaks represent intermediate similarity.

**
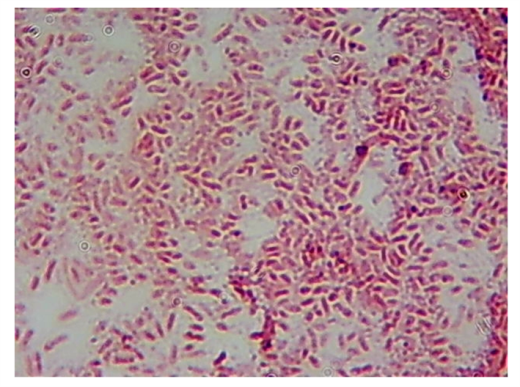
**

**Figure S7.** Gram staining of bacteria from the same clinical sample examined by the new method.

**References**

[1] J.H. Kang, M. Super, C.W. Yung, R.M. Cooper, K. Domansky, A.R. Graveline, T. Mammoto, J.B. Berthet, H. Tobin, M.J. Cartwright, A.L. Watters, M. Rottman, A. Waterhouse, A. Mammoto, N. Gamini, M.J. Rodas, A. Kole, A. Jiang, T.M. Valentin, A. Diaz, K. Takahashi, D.E. Ingber, An extracorporeal blood-cleansing device for sepsis therapy, Nat. Med. 20(10) (2014) 1211-6.

[2] K. Takahashi, W.C. Chang, M. Takahashi, V. Pavlov, Y. Ishida, L. La Bonte, L. Shi, T. Fujita, G.L. Stahl, E.M. Van Cott, Mannose-binding lectin and its associated proteases (MASPs) mediate coagulation and its deficiency is a risk factor in developing complications from infection, including disseminated intravascular coagulation, Immunobiology 216(1-2) (2011) 96-102.

[3] J. Sun, H. Shi, Y. Xue, W. Cheng, M. Yu, C. Ding, F. Xu, S. Yu, Releasing bacteria from functional magnetic beads is beneficial to MALDI-TOF MS based identification, Talanta 225 (2021) 121968.
